# Combined Endoscopy-Assisted Muscle-Sparing Latissimus Dorsi Flap Harvesting with Lipofilling Enhancement as a New Volume Replacement Technique in Breast Reconstruction

**DOI:** 10.1155/2022/7740439

**Published:** 2022-01-31

**Authors:** Yasser S. Ahmed, Walid M. Abd El Maksoud

**Affiliations:** ^1^Experimental Surgery Department, Medical Research Institute, Alexandria University, Alexandria, Egypt; ^2^Surgery Department, Faculty of Medicine, King Khalid University, Abha, Saudi Arabia

## Abstract

**Introduction:**

This study evaluated the feasibility and patient satisfaction of combined endoscopy-assisted muscle-sparing latissimus dorsi flap harvesting, with lipofilling enhancement for skin-preserving mastectomy.

**Methods:**

This is a prospective study that included 21 female patients with small breasts (cup size A-B), subjected to skin-preserving mastectomy as a management of breast cancer. Combined endoscopy-assisted muscle-sparing latissimus dorsi flap harvesting with lipofilling enhancement was performed for immediate breast reconstruction. Patients were followed up for early and late postoperative complications including recurrence for at least 24 months. Postoperative patient satisfaction was assessed using the Kyungpook National University Hospital breast reconstruction satisfaction questionnaire.

**Results:**

The study included 21 female patients with a mean age of 42.10 ± 8.46 years. Patients were followed up for 26.67 ± 3.38 months. The procedure was successful in all patients with a mean duration of 172.05 ± 28.22 minutes. Local recurrence was encountered in one patient (4.67%). Eighteen patients declared their satisfaction 6 months after the operation, while two patients were satisfied only after the second session of lipofilling. The overall postoperative patient satisfaction was 95.24%. The majority of the patients (93.3%) who underwent NSM surgery were satisfied, while only two-thirds (66.6%) of the patients who underwent SSM surgery were satisfied.

**Conclusions:**

Combined endoscopy-assisted muscle-sparing latissimus dorsi flap harvesting with lipofilling enhancement seems to be a feasible and encouraging technique for the volume adjustment of small breasts, especially in nipple-sparing mastectomy. It leaves a minor back scar and has an acceptable rate of postoperative complications. The procedure showed high postoperative patient satisfaction.

## 1. Introduction

Breast cancer (BC) is the most frequently diagnosed cancer in women worldwide, representing 25.4% of the total number of new cases diagnosed in 2018 [1]. It is estimated that, in 2021, the number of new cases of invasive breast cancer that are expected to be diagnosed in women in the United States will be 281,550, and 43,600 women will die from breast cancer [2].

Nowadays, women diagnosed with breast cancer for the first time are offered a range of treatment options within a multidisciplinary setting [3]. The multidisciplinary team approach has been employed internationally for decades, aiming to create collaborative decision making and benefitting from all clinical experience from multiple specialties on single-patient cases in a systematic fashion [4].

Modern surgical treatment options for breast cancer are based on oncoplastic techniques to achieve both superior cosmetic and cancer treatment outcomes [5]. In addition, careful preoperative planning with other specialists such as radiologists, pathologists, and oncologists is an integral part of the oncoplastic approach in the modern management of breast cancer [6].

Although multiple techniques for breast construction have been created, there is still no consensus regarding the safest method with the best long-term results. Achieving oncological safety and patient satisfaction for superior cosmetic outcomes has been challenging up to date [7].

The latissimus dorsi (LD) muscle flap is one of the methods of breast reconstruction after skin-reducing mastectomy (nipple-sparing mastectomy and skin-sparing mastectomy) [8]. Despite its widespread use, there are two limitations of its current approach for improving the breast shape. The first is the large remaining donor site scar after flap surgery for breast reconstruction, and the second is the small volume of the muscle flap which often requires augmentation with a breast implant to achieve an acceptable esthetic result [9]. The use of implants, however, is not without risks. The use of prosthetic-based breast reconstruction was reported to be associated with capsular contracture, infection, implant rupture, and extrusion, and the potential for reoperation to exchange the implant after the useful lifetime of the device has passed [10].

Autologous fat transplantation, also known as autologous fat grafting (AFG) or lipofilling, is the process of relocating autologous fat to change the shape, volume, consistency, and profile of tissues [11]. In 2016, it was reported that lipofilling does not increase the risk of recurrence in a cancerous breast [12]. Furthermore, LD flap enhancement by lipofilling as an immediate breast reconstruction technique for skin-preserving mastectomy was reported to be a successful and safe technique for the achievement of autologous breast reconstruction with high postoperative patient satisfaction [13].

Limited space and difficult angles were the main obstacles hindering endoscopic breast surgery [14, 15]. However, endoscopic surgery for harvesting the LD flap has been reported more frequently because securing space is easier in the LD cavity than in the breast [16–18]. Endoscopy-assisted LD flap harvest may eliminate the problem of scarring of the donor site, but the small size of the LD flap requires augmentation to be useful in proper reconstruction of the breast.

Therefore, the aim of our study was to evaluate the feasibility and patient satisfaction of combined endoscopy-assisted muscle-sparing LD flap harvesting with lipofilling enhancement for skin-preserving mastectomy.

## 2. Materials and Methods

The study had a prospective design. It included female patients who were admitted to the Surgery Department of the Medical Research Institute Hospital, Alexandria University, from January 2018 to January 2019. The patients underwent combined endoscopic LD flap harvesting and enhancement by lipofilling as an immediate breast reconstruction technique, for skin-preserving mastectomy for the oncoplastic management of their condition.

Inclusion criteria were female patients ≥18 years, who had small- and moderate-sized breasts (cup size A-B), with breast cancer clinically staged as I, II, or IIIA. They were not candidates for breast-conserving surgery and were eligible for skin-preserving mastectomy.

The exclusion criteria entailed patients with bleeding disorders, smokers, patients with inflammatory BC, and patients with large breasts (C-D).

### 2.1. Preoperative Work-Up

All patients were subjected to a complete assessment of their history, including age, family history, and use of oral contraceptive pills or hormonal intrauterine devices. Thorough clinical examination of both breasts was performed, in addition to radiologic investigations in the form of mammography and/or ultrasonography and a metastatic radiologic work-up. All patients were subjected to fine-needle aspiration cytology (FNAC) or a true-cut biopsy and histopathological examination to confirm their diagnosis.

Written informed consent was taken from all patients regarding the steps of the operation and the potential complications of AFG.

### 2.2. Operative Work-Up

**Figure 1:**
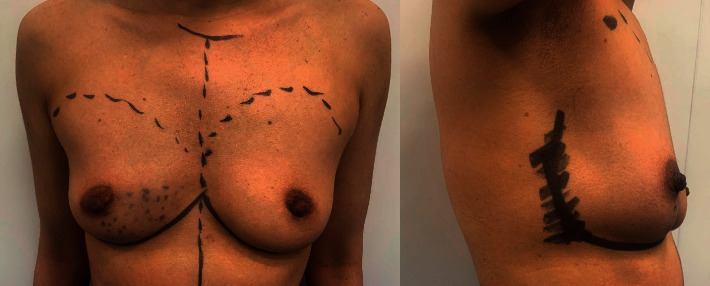
Preoperative drawing of the patient.

Drawing of the patients was performed while the patients were standing (Figure 1). All patients were operated by the same team of surgeons who are experts in the field of oncoplastic breast surgery.

### 2.3. Mastectomy Technique

This was achieved by skin-preserving mastectomy, i.e., either skin-sparing mastectomy or nipple-sparing mastectomy with preservation of the noninvolved skin or nipple. The axilla was managed by axillary clearance through a separate incision or a sentinel lymph node biopsy. The volume of the excised breast tissue (V1) was assessed by the fluid displacement method to determine the exact volume required for replacement.

### 2.4. Endoscopic-Assisted Latissimus Dorsi Flap (EALDF)

**Figure 2:**
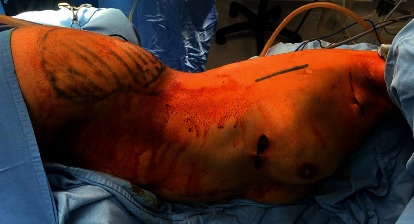
Putting of the patient in the lateral position for endoscopic LD flap harvesting.

**Figure 3:**
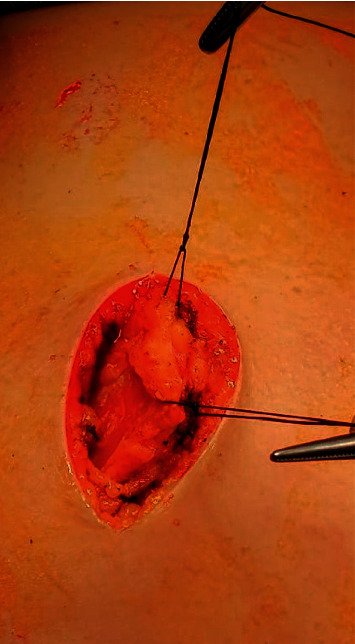
Identification of the anterior border of the LD muscle.

**Figure 4:**
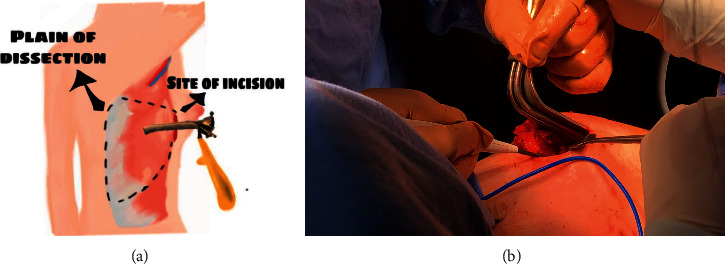
(a) A diagram showing the steps of the procedure; (b) dissection of the outer surface of the LD muscle from the subcutaneous tissue using electric diathermy with a long pen.

**Figure 5:**
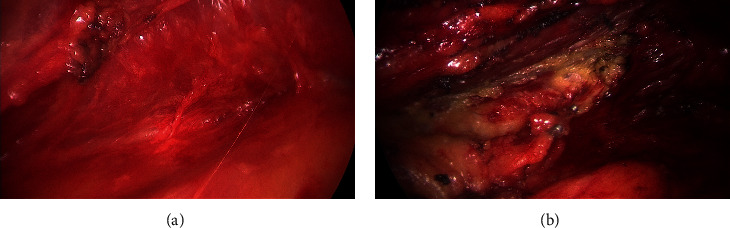
(a) Blunt dissection of the under surface of the LD muscle; (b) clipping of the perforator.

**Figure 6:**
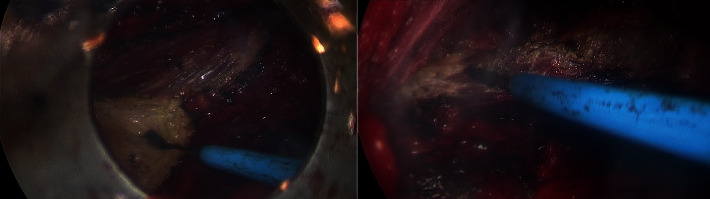
Endoscopic cutting of the posterior border of the LD muscle.

**Figure 7:**
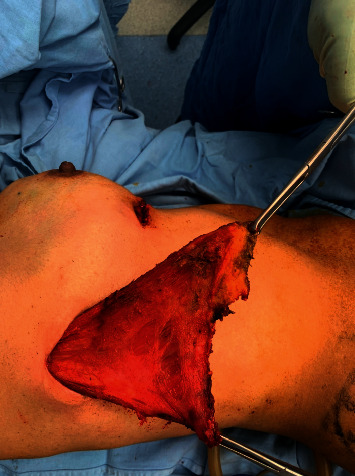
Elevation of the LD muscle till the neurovascular bundle.

**Figure 8:**
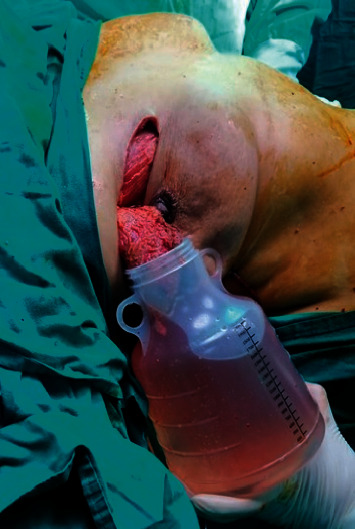
Measurement of the volume of the LD by fluid displacement.

The patient was placed in the lateral position with the arm elevated to 90 degrees (Figure 2). The procedure was started by creating a vertical incision 4–6 cm at the midaxillary line at the level of the nipple, which is the most accessible site to obtain all parts of the LD muscle. Dissection of the skin and subcutaneous tissue was performed with the detection of the anterior border of the LD muscle (Figure 3). The endoscopic illuminating breast retractor (sculpo endoscopic retractor with a channel for endoscopes, Model nm-di-786813, New Med Supplies, Pakistan) was introduced from the wound; thereafter, dissection of the outer surface of the LD muscle from the subcutaneous tissue was performed using electric diathermy with a long pen (Figure 4). This was followed by a blunt dissection of the inner surface of the muscle and clipping all perforators (Figure 5). The lower border of the LD muscle was cut, followed by a separation of the posterior border of the LD muscle (Figure 6) and an elevation of the LD muscle till its neurovascular pedicle (Figure 7). We detected the volume of the LD flap (V2) by the fluid displacement method using a cylinder with saline (Figure 8). The flap was located within the skin envelope of the breast through a tunnel in the axilla.

Preparation of the donor site (abdomen or lateral aspect of the thigh) was performed by injection of tumescent saline followed by fat harvesting and centrifugation. The lipofilling was prepared using the Coleman technique [19].

**Figure 9:**
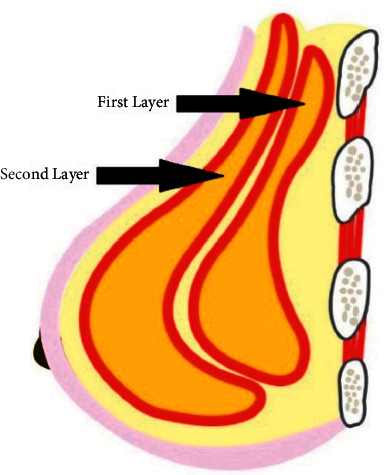
A diagram showing the layers of autologous fat graft injection; the first layer includes the pectoralis major and serratus anterior, and the second layer includes LD muscle.

Subtraction of the volume of the LD flap from the volume of the excised specimen (V1-V2) resulted in the estimated guide volume (V3) for the amount of autologous fat needed for enhancement. Injection of the fat graft was performed in two layers. The first layer (pectoralis major and serratus anterior) and the second layer are in the LD flap in different planes (Figure 9). The amount of fat injection was V3 ± 25%. The signs to stop injection of the fat were congestion of the muscle or back flow of the fat from the muscle.

Two negative suction drains were added separately, one in the LD bed and the other for the breast and axilla. Gentle pressure on the LD site was applied using a bandage.

### 2.5. Postoperative Work-Up

Patients were discharged on the second postoperative day provided the pain was tolerable and no complications were observed. Negative suction drainage was removed in the follow-up visits when the amount of drained fluid was less than 50 ml.

**Figure 10:**
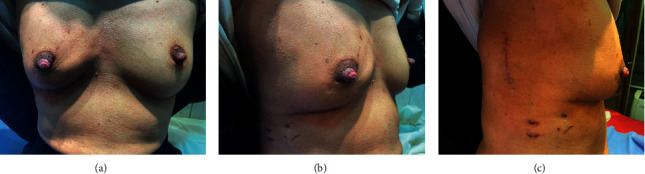
Postoperative picture of the patient: (a) anterior view, (b) oblique view, and (c) lateral view.

Patients were followed up at 3, 6, 12, and 24 months for diagnosing possible early and late postoperative complications and for the detection of an oncological recurrence (Figure 10).

Patient satisfaction regarding the outcome was assessed utilizing the Kyungpook National University Hospital (KNUH) breast reconstruction satisfaction questionnaire [20] that was provided to all patients 6 months after surgery. Patients who showed dissatisfaction were offered other sessions of lipofilling, and their satisfaction was reassessed 3 months after the last session.

### 2.6. Outcomes

#### 2.6.1. Primary End Points

- Feasibility of the technique was determined by the operative nurse by recording the incidence of turnover of the open technique and the operative time during the operation
- Patient satisfaction was recorded by asking the patients to fill the Kyungpook National University Hospital (KNUH) breast reconstruction satisfaction questionnaire [20] during the follow-up visits at 6 months after surgery

#### 2.6.2. Secondary End Points

- Early postoperative complications of the breast, the back, and the donor site that were detected during the follow-up visits within the first month, postoperatively, via examination by the surgeon
- Late postoperative complications that were detected during the follow-up visits after 3, 6, 12, and 24 months, postoperatively, through examination by the surgeon and/or the radiologic investigations

### 2.7. Statistical Analysis

The statistical analysis of the data was performed using the Statistical Package for the Social Sciences (SPSS version 25; SPSS Inc., Chicago, Illinois, USA). Descriptive statistics were applied (frequency and percentage for categorical variables and mean and SD for quantitative variables). Fisher's exact test and the Mann–Whitney *U* test were used to test significance of differences for qualitative and quantitative data, respectively. Statistically significant differences were considered at *P* values equal to or less than 0.05.

### 2.8. Ethical Approval

The research was approved by the Institutional Research Board of the College of Medicine, Alexandria University (IRB 00012098), and precautions were taken to conceal the identity of patients. Items of the PROCESS checklist [21] were fulfilled.

## 3. Results

The study included 21 female patients who fulfilled the inclusion criteria. The patients had a mean age of 42.10 ± 8.46 years and a mean body mass index of 29.09 ± 4.08 kg/m^2^. The demographic and preoperative clinical data of the patients are shown in Table 1.

Nipple-sparing mastectomy was performed in 15 patients (71.43%) compared with skin-sparing mastectomy that was performed in 6 patients (28.57%). The mean time of the operation was 172.05 ± 28.22 minutes. The mean amount of AFG was 236.67 ± 65.98 ml, which represented 124.40 ± 3.97% of the V3 volume. The operative data of the patients are shown in Table 2. The mean period of follow-up was 26.67 ± 3.38 months.

Regarding late postoperative complications, local recurrence was encountered in one patient (4.76%) 19 months after the operation, and the patient was offered mastectomy. Early and late postoperative complications of the surgery are shown in Table 3.

The majority of the patients (93.3%) who underwent NSM surgery were satisfied, while only two-thirds (66.6%) of the patients who underwent SSM surgery were satisfied. Patients who underwent NSM showed significant better satisfaction compared to patients who underwent SSM. Furthermore, analysis of the elements of the questionnaire revealed that patients who underwent NSM showed significant better satisfaction score compared to patients who underwent SSM regarding the following elements, size of the reconstructed breast, scar of the reconstructed breast, self-confidence, sexual attraction, and overall satisfaction. There was no significant difference (*P*=0.465) in the satisfaction score between patients who received postoperative radiotherapy (4.02 ± 0.69) and patients who did not receive radiotherapy (4.33 ± 0.11). Three patients in this study were dissatisfied, 2 of them underwent SSM and one patient underwent NSM. Two patients underwent another session of lipofilling, while one patient underwent two more sessions of lipofilling. Two out of the three patients expressed their satisfaction in the questionnaire performed 3 months after the last session of lipofilling, raising the overall satisfaction to 95.24%. Satisfaction among our patients is shown in Tables 4 and 5.

## 4. Discussion

Nowadays, skin-preserving mastectomy is considered the best esthetic solution for cases of multicentric early breast cancer without fear of catastrophic mastectomy options for patients, especially young patients [22, 23]. Apparently, the benefit of skin-sparing mastectomy is the esthetic outcome. However, the benefits extend to include better postoperative psychological status of the patient and avoid the breast cancer stigma and mastectomy [24].

Immediate reconstruction after skin-preserving mastectomy is considered as a requisite for the comprehensive care of patients with breast cancer [25, 26]. Volume replacement can be achieved by either implant-based volume replacement or autologous flap-based volume replacement. Implant-based volume replacement, either for immediate or delayed reconstruction, has many disadvantages, such as capsular contracture, infection, and exposure in addition to its cost [27, 28].

In this study, we chose to use the LD flap as an autologous flap-based reconstruction. The autologous-based reconstruction as a pedicle flap (TRAM and LD flap) or free flap (DIEP flap) has many advantages. Autologous reconstruction feels more natural and lasts a lifetime. In addition, being composed of natural tissues, it can better withstand the radiotherapy [29]. Santosa et al. [30] reported that, after 2 years of performing reconstruction, patients who underwent autologous reconstruction were more satisfied with their breasts and had greater psychosocial well-being and sexual well-being than those who underwent implant reconstruction.

Our choice for the LD flap in the current study was based on the fact that it was safe and effective with good esthetic outcomes [13]. However, the main disadvantage of this technique is the donor site scar, which was the main point of concern for our patients.

Endoscopic harvesting of the LD muscle was proposed to be the solution for the back scar problem. Nevertheless, it failed to gain popularity because of difficulties in maintaining the optic cavity and thorax anatomic curvature, in addition to the prolonged time of the procedure [31]. Consequently, some studies reported that the technique is difficult and uncommon [32, 33]. The technique was abandoned by Vasconez [34] in his study.

The endoscopy-assisted LD flap harvesting technique was easily mastered by the surgeons in our study as laparoscopic skills constituted a constant part of their surgical training program. However, due to the long period spent in not utilizing their laparoscopic skills as breast surgeons, in the beginning of their learning curve, the duration of the technique was longer and lasted 180 minutes. At the end of the learning curve, the duration was markedly decreased to 80 minutes.

Our technique was different compared with the technique described by Lee et al. [17], as we used a breast retractor with an attached 10 mm camera. It offered the advantage of better camera handling through the narrow space and freed one hand of the assistant. In addition, we used the long diathermy pen instead of the laparoscope spatula owing to our familiarization with the instruments, as general surgeons. This led to a better handling of the tissue. We combined both open and endoscopic skills as we used direct visualization of some steps as separation of the outer surface of the muscle and anterior border identification, while using the camera in difficult parts of visualization as inner surface and posterior and inferior borders of the muscle.

In this study, after harvesting the muscle through our endoscopic-assisted technique, the main disadvantage was the small volume of the muscle compared with the conventional musculocutaneous LD flap. The mean volume of the muscle in our series was 228.76 ± 5756 ml, while Ahmed et al. [13] reported that the mean volume of the LD flap was 446.40 ± 70.89 ml. This could be attributed to the added volume of the skin paddle and subcutaneous tissue present in the musculocutaneous LD flap. However, we could have compensated for the smaller volume of the LD by performing the lipofilling technique for enhancement of the volume in the form of a two-layer method, which would have given us more surface area for fat injection. Furthermore, it increased the fat uptake and reduced the complications of injection of large amounts of fat in the narrow area. The percentage of the injected fat compared with V3 was 124.40 ± 3.97%, as compared with 106.80 ± 13.90% that was reported in the conventional musculocutaneous LD flap [13].

In the current study, 18 patients (85.7%) received PMRT while only 3 patients (14.3%) did not receive PMRT. There were no significant differences between the satisfaction scores between those who received and those who did not receive PMRT. Berthet et al. [35] evaluated breast consistency of 154 patients who underwent LD flap reconstruction. They reported that 93.1% of patients rating outcomes as “very good” or “good” in the irradiated group compared to the 82.7% in the nonirradiated group; however, the difference was not statistically significant. Their results, in addition to our results, may support that the autologous LD flap can withstand the radiotherapy even if it was enhanced by fat graft.

Regarding the postoperative patient satisfaction, the overall result of our patients including those who were satisfied 6 months postoperatively and those who were satisfied after they had another lipofilling session was 95.24%. These results are better than what was reported for conventional LD flap with AF enhancement or by endoscopy-assisted LD flap without AF enhancement [13, 17]. Nevertheless, we have to stress that our results refer to small-sized breasts (cup size A-B) and not large breasts that may be suitable in other techniques. Furthermore, satisfaction of patients who underwent NSM was significantly better than that of those who underwent SSM. This was clear in the total satisfaction score and in several elements of the score. Although this study revealed that the technique of endoscopy-assisted LD flap with AF enhancement is feasible for patients who underwent SSM, yet further studies regarding the patients' satisfaction with larger number of patients may be required before recommending this technique for reconstruction after SSM.

Regarding patients' satisfaction, previous studies utilizing endoscopy-assisted LD flap harvest for breast reconstruction [36, 37] have reported satisfactory results regarding the back scar. However, satisfaction towards the reconstructed breast was moderate to good due to the small volume of LD muscle or asymmetry with the other breast. In our study, this disadvantage was ameliorated using lipofilling to increase the volume of muscle and consequently improving the esthetic results of our patients. This has improved satisfaction among our patients towards their newly reconstructed breast.

Our study is limited by being a single-center study. A large sample size may be required with longer period of follow-up to support our results.

## 5. Conclusions

Combined endoscopy-assisted muscle-sparing latissimus dorsi flap harvesting with lipofilling enhancement seems to be a feasible and encouraging technique for the volume adjustment of small breasts, especially in nipple-sparing mastectomy. It leaves a minor back scar and has an acceptable rate of postoperative complications. The procedure showed high postoperative patient satisfaction.

## Figures and Tables

**Table 1:** Demographic and preoperative clinical data of the patients.

	Studied group (*n* = 21)
Age (years)	
Range	28–56
Mean	42.10
SD	8.46
BMI (kg/m^2^)	
Range	28–37
Mean	29.90
SD	4.08
Affected side	
Right	8 (38.10%)
Left	13 (61.90%)
Clinical staging, *n* (%)	
I	5 (23.81%)
IIA	6 (28.57%)
IIB	5 (23.81%)
IIIA	5 (23.81%)
Histopathological type	
Infiltrative ductal carcinoma	16 (76.19%)
Infiltrative lobular carcinoma	4 (19.05%)
Mixed type	1 (4.76%)

**Table 2:** Operative data of the patients.

	Studied group (*n* = 21)
Operative time (minutes)	
Range	118–215
Mean	172.05
SD	28.22
Type of the surgical procedure, *n* (%)	
Nipple-sparing mastectomy	15 (71.43%)
Skin-sparing mastectomy	6 (28.57%)
Type of the surgical management of the axilla, *n* (%)	
Axillary clearance	13 (61.90%)
Sentinel lymph node biopsy	8 (38.10%)
Volume of the excised specimen (ml)	
Range	250–545
Mean	419.76
SD	78.31
Volume of latissimus dorsi (ml)	
Range	115–330
Mean	228.76
SD	57.56
Volume of injected fat (ml)	
Range	160–400
Mean	236.67
SD	65.98
Percentage of injected fat compared to V3^*∗*^ (%)	
Range	118.34–130.43
Mean	124.40
SD	3.97
Type of postoperative adjuvant therapy, *n* (%)	
Hormonal therapy only	1 (4.76%)
Hormonal therapy and chemotherapy	2 (9.52%)
Hormonal therapy and radiotherapy	6 (28.57%)
Radiotherapy and chemotherapy	7 (33.33%)
Hormonal therapy, radiotherapy, and chemotherapy	5 (23.81%)

^*∗*^V3 = volume of the excised specimen−volume of LD.

**Table 3:** Early and late postoperative complications of the surgery.

	Studied group (*n* = 21)
Follow-up period (months)	
Range	24–34
Mean	26.67
SD	3.38
Early postoperative complications of the breast, *n* (%)	
Partial skin necrosis	1 (4.76%)
Seroma	10 (47.62%)
Ecchymosis	3 (14.29%)
Wound infection	2 (9.52%)
Early postoperative complications of the back, *n* (%)	
Seroma	3 (14.29%)
Ecchymosis	0 (0.0%)
Hematoma	0 (0.0%)
Early postoperative complications of the donor site, *n* (%)	
Hematoma	0 (0.0%)
Ecchymosis	8 (38.10%)
Late postoperative complications, *n* (%)	
Recurrence	1 (4.76%)
Oil cyst	0 (0.0%)
Macrocalcifications	6 (28.57%)
Complete flap loss	0 (0.0%)

**Table 4:** Comparison between NSM and SSM regarding patients' satisfaction using the KNUH questionnaire.

			Type of surgery		Total	*P* value
			NSM	SSM		
Satisfaction	Satisfied	Number	14	4	18	
		%	93.9	66.7		
	Dissatisfied	Number	1	2	3	
		%	6.7	33.3		
Total		Number	15	6	21	
		%	100.0	100.0	100.0	0.184

*P* < 0.05 is significant.

**Table 5:** Comparison between patients who underwent NSM and patients who underwent SSM regarding the KNUH questionnaire scores and its elements.

Question number	Questions	NSM (*n* = 15), mean ± SD	SSM (*n* = 6), mean ± SD	Total (*n* = 21), mean ± SD	*P* value
1	Symmetry of the breast	4.13 ± 0.74	3.50 ± 1.05	3.95 ± 0.86	0.134
2	Size of my reconstructed breast	4.27 ± 0.59	3.50 ± 1.05	4.04 ± 0.80	0.04^*∗*^
3	Shape of my reconstructed breast	4.00 ± 0.76	3.50 ± 1.22	3.86 ± 0.91	0.266
4	Feel to touch my reconstructed breast	4.13 ± 0.64	4.16 ± 0.75	4.14 ± 0.65	0.927
5	Pain in my reconstructed breast	4.46 ± 0.64	4.50 ± 0.84	4.48 ± 0.68	0.906
6	Scar of my reconstructed breast	4.40 ± 0.51	3.16 ± 0.75	4.05 ± 0.80	0.001^*∗*^
7	Self-confidence	4.33 ± 0.82	3.50 ± 0.84	4.10 ± 0.89	0.05^*∗*^
8	Sexual attraction	4.20 ± 0.56	3.00 ± 1.26	3.86 ± 0.96	0.006^*∗*^
9	Overall satisfaction	4.33 ± 0.62	3.50 ± 0.84	4.1 ± 0.77	0.021^*∗*^
	Total	4.25 ± 0.44	3.59 ± 0.86	4.04 ± 0.64	0.030^*∗*^

^*∗*^Statistically significant (*P* value ≤0.05).

## Data Availability

Data can be obtained from the corresponding author on reasonable request.
